# Non-Homogeneous Fractal Hierarchical Weighted Networks

**DOI:** 10.1371/journal.pone.0121946

**Published:** 2015-04-07

**Authors:** Yujuan Dong, Meifeng Dai, Dandan Ye

**Affiliations:** Faculty of Science, Jiangsu University, Zhenjiang 212013, Jiangsu, China; UNMdP-CONICET, ARGENTINA

## Abstract

A model of fractal hierarchical structures that share the property of non-homogeneous weighted networks is introduced. These networks can be completely and analytically characterized in terms of the involved parameters, i.e., the size of the original graph *N_k_* and the non-homogeneous weight scaling factors *r*_1_, *r*_2_, · · · *r_M_*. We also study the average weighted shortest path (AWSP), the average degree and the average node strength, taking place on the non-homogeneous hierarchical weighted networks. Moreover the AWSP is scrupulously calculated. We show that the AWSP depends on the number of copies and the sum of all non-homogeneous weight scaling factors in the infinite network order limit.

## Introduction

Many complex systems can be represented as graphs or networks, where nodes represent the elementary units of a system and links standing for the interactions between the nodes. In recent years, we observed an increasing number of papers [1–6] where authors proposed a new point of view by constructing networks exhibiting scale-free and hierarchical structures by adapting ideas taken from fractal construction; e.g. Koch curve or Sierpinski gasket. Weighted networks represent the natural framework to describe natural, social, and technological systems, in which the intensity of a relation or the traffic between elements is an important parameters [7, 8]. In general terms, weighted networks are extension of networks or graphs [9, 10], in which each edge between nodes i and j is associated with a variable *w*_*ij*_, called the weight. In the case of the world-wide airport network the weight *w*_*ij*_ of an edge linking airports i and j represents the number of available seats in flights between these two airports. The inspection of the weights shows that the average numbers of seats in both directions are identical *w*_*ij*_ = *w*_*ji*_ for an overwhelming majority of edges. Many real traffic systems are better represented and understood as weighted networks, where nodes and links are assigned to some weight values representing their physical properties such as capacity and delay. In a weighted aviation network, link weight can denote the annual volume of passengers traveling between two airports. A realistic form of networks are weighted networks, where each link is assigned to a weight value to denote a physical property of interest, e.g., the length of a road, and similarly, each node is assigned to a strength to represent, for instance, the computational capacity of an Internet router. In addition, individual links (and nodes) on weighted networks are essentially different.

In complex networks, the average degree and the average node strength are calculated to analyze the properties or characteristics of the network. The degree *k*_*i*_ of a node *i* is the number of edges connected to it. Closely related to the density of a network is the average degree, $<k>=\frac{\sum_{i=1}^{N}k_{i}}{N}$, where *k*_*i*_ is the degree of the node *i* and N is the number of nodes in complex networks. A more significant measure of the network properties in terms of the actual weights is obtained by extending the definition of vertex degree*k*_*i*_ in terms of the node strength *w*_*i*_, defined as *w*_*i*_ = ∑_*j* ∈ *ν*(*i*)_ *w*_*ij*_, where the sum index *j* runs over the set *ν*(*i*) of neighbors of the node *i*. This quantity measures the strength of vertices in terms of the total weight of their connections. To shed more light on the relationship between the vertices strength and degree, A. Barrat et al. [11] have obtained *w*_*i*_ = ⟨*w*⟩*k*_*i*_.

Recently, attention also has been given to the average weighted shortest path (AWSP) on weighted networks in communication networks. Knowledge on AWSP is relevant to the design and engineering of real systems such as air transport networks and highway networks, for example it exhibits how to deploy network resources, how to route traffic efficiently and how to mitigate congestion.

It is well known that one of the most amazing and interesting feature of fractals is their self-similarity, namely looking at all scales we can find conformal copies of the whole set. Starting from this property which can provide rules to build up fractals as fixed points of Iterated Function Systems [12, 13], IFS for short, non-homogeneous weighted networks is introduced. Non-homogeneous weighted networks are completely characterized by two main parameters: the number of copies *M*+1 > 1 and the scaling factor 0 < *r* < 1 of the IFS. Let us fix some positive real numbers *r*_1_,*r*_2_,⋯,*r*_*M*_ ∈ (0,1) and a positive integer *M* > 1 and let us consider an non-homogeneous weighted network *G* composed of *N*_*k*_ nodes, one of which has been labeled attaching node and denoted by the hub of *G* ^0^. Non-homogeneous weighted networks also represent an explicitly computable model for the renormalization procedure recently applied to complex networks [14–16].

In this paper, we study the average degree, the average node strength and average weighted shortest path on a class of non-homogeneous fractal hierarchical weighted networks. Firstly, we introduce the non-homogeneous fractal hierarchical weighted networks. Secondly, our results show that the average degree of these networks is very close to a constant controlled by the number of copies and the average node strength goes to zero as iteration number increases. Finally, we reveal that the average weighted shortest path is controlled by the number of copies and all non-homogeneous weight scale factors as the iteration number goes to infinity.

## Results and Discussion

### Non-homogeneous weighted hierarchical modular networks

In order to construct our model, we have referred to a large number of references. The number of the initial nodes is 5 in [1] and un-weight in [1, 2]. By contrast, we may expand the number of initial nodes to *M*+1 and add a weight to each edge of the network. So the results in [1, 2] are just special cases. Recently, Carletti [4] proposed a non-homogeneous weighted fractal network aiming to construct weighted networks with some a priori prescribed topology depending on two kinds of parameters: the number of copies and the scaling factors. Enlightened by Carletti’s network, we will build weighted hierarchical networks depending on M scaling factors (i.e., *r*_1_,*r*_2_,⋯,*r*_*M*_).

Now let us introduce a new model for the weighted hierarchical networks in our paper, which is controlled by a positive integer *M* and *M* real numbers *r*_1_,*r*_2_,⋯,*r*_*M*_ ∈ (0,1) with a modular structure which can be constructed in an iterative way. We denote by *G*_*k*_ the network model after *k* (*k* ≥ 1) iterations.

**Fig 1:**
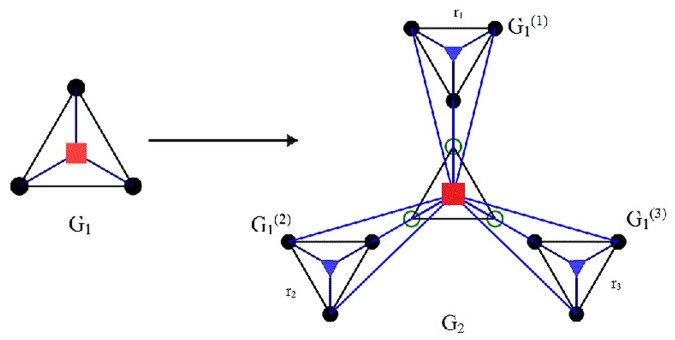
(color online) The iterative construction process from *G*_1_ to *G*_2_ for the case of *M* = 3. *G*_1_ includes 4 initial nodes and 6 edges with unit weight; in *G*_2_, $G_{1}^{\left(i\right)}$ is a copy of *G*_1_ scaled by a factor *r*_*i*_(*i* = 1,2,3). The 3 external nodes of each replica $G_{1}^{\left(i\right)}(i=1,2,3)$ to the root of the original *G*_1_ through edges of unitary weight.

**Fig 2:**
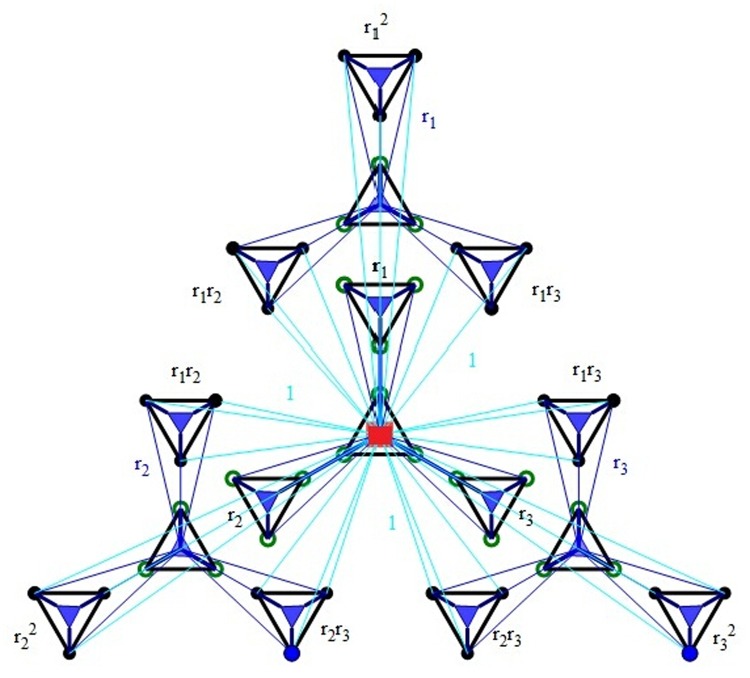
(color online) Classification of nodes in network *G*_3_ for the case of *M* = 3. The filled circles, open circles, full square and triangles represent peripheral nodes, locally peripheral nodes, hub nodes and locally hub nodes, respectively. In *G*_3_, $G_{2}^{\left(i\right)}$ is a copy of *G*_2_ scaled by a factor *r*_*i*_(*i* = 1,2,3). The 9 external nodes of each replica $G_{2}^{\left(i\right)}(i=1,2,3)$ to the root of the original *G*_1_ through edges of unitary weight.

(1) Initially *k* = 1, the network *G*_1_ consists of a central node, called the hub (root) node *A*, and *M* peripheral (external) nodes with *M* ≥ 2. All these initial *M*+1 nodes are fully connected to each other, forming a complete graph. Each of the edges carries a standard initial weight *w* = 1.

(2) At the second generation (*k* = 2), we generate *M* copies of *G*_1_ whose weighted edges have been scaled respectively by a factor *r*_1_,*r*_2_,⋯,*r*_*M*_. The *M* copies are labeled as $G_{1}^{\left(1\right)},G_{1}^{\left(2\right)},\cdots ,G_{1}^{\left(M\right)}$. And we connect the *M* external nodes of each replica $G_{1}^{\left(i\right)}(i=1,2,\cdots ,M)$ to the root of the original *G*_1_ through edges of unitary weight. The original *G*_1_ in *G*_2_ has label $G_{1}^{\left(0\right)}$. The hub of the original $G_{1}^{\left(0\right)}$ and *M* ^2^ peripheral nodes in the replicas $G_{1}^{\left(i\right)}(i=1,2,\cdots ,M)$ become the hub and peripheral nodes of *G*_2_, respectively. The hub node in *G*_2_ also has label *A*. The process is described in Fig. 1.

(3) Suppose one has *G*_*k*−1_, the next generation network *G*_*k*_ can be obtained from *G*_*k*−1_ by adding *M* replicas of *G*_*k*−1_, whose weighted edges have been scaled respectively by a factor *r*_1_,*r*_2_,⋯,*r*_*M*_. The *M* copies are labeled as $G_{k-1}^{\left(1\right)},G_{k-1}^{\left(2\right)},\cdots ,G_{k-1}^{\left(M\right)}$. And their external nodes are linked to the hub of the original *G*_*k*−1_ through edges of unitary weight. The original *G*_*k*−1_ in *G*_*k*_ has label $G_{k-1}^{\left(0\right)}$. In *G*_*k*_, its hub is the hub of the $G_{k-1}^{\left(0\right)}$, and its external nodes are composed of all the peripheral nodes of the $G_{k-1}^{\left(i\right)}(i=1,2,\cdots ,M)$. The hub node in *G*_*k*_ always has label *A*. Moreover, hub node *A* is always the same, that there are *N*_*k*_ (order of the network) external nodes at each stage. The classification of nodes is described in Fig. 2.

(4) Repeating the replication and connection steps, we obtain the weighted hierarchical modular networks.

### Average degree and average node strength

In *G*_*k*_, the number of nodes, often called order of the network denoted as *N*_*k*_, is

$$\begin{array}{c} N_{k}={\left(M+1\right)}^{k}, \end{array}$$

and the number of edges at the time of *k* is

$$\begin{array}{c} E_{k}=\frac{1}{2}M{\left(M+1\right)}^{k}+M^{2}{\left(M+1\right)}^{k-1}-M^{k+1}. \end{array}$$

Therefore, the average degree is asymptotically given by

$$\begin{array}{c} \frac{E_{k}}{N_{k}}\to \frac{M(3M+1)}{2(M+1)},\;\;k\to \infty . \end{array}$$

when *k* increases to infinity.

Denote by $w_{i}^{k}$ the weighted degree of node *i* at iteration *k*, we also call it node strength [17] and the node strength satisfies the following formula $w_{i}^{k}=\sum_{j}w_{ij}^{\left(k\right)}$, where $w_{ij}^{\left(k\right)}$ stands for the weight of the edge (*ij*) ∈ *G*_*k*_. According to the recursive construction in our weighted hierarchical networks, we can explicitly compute the total node strength, $w_{k}=\sum_{i}w_{i}^{\left(k\right)}$, and easily show that

$$\begin{array}{c} w_{k}={\left(1+r\right)}^{k-1}M\left(M+1\right)+M^{k}\left[1+\frac{1+r}{M}+\cdots +{\left(\frac{1+r}{M}\right)}^{k-2}\right]\\ =M\left(M+1\right){\left(1+r\right)}^{k-1}+\frac{M^{2}{\left(1+r\right)}^{k-1}-M^{k+1}}{1+r-M}, \end{array}$$

where $r=\sum_{j=1}^{M}r_{j}$. In view of *r*_*j*_ < 1(*j* ∈ *i*,2,⋅⋅⋅,*M*), it trivially follows that *r* < *M*, so we can conclude the average node strength goes to zero when *k* increases to infinity:

$$\begin{array}{c} \frac{w_{k}}{N_{k}}\to 0,\;\;k\to \infty . \end{array}$$

### Average weighted shortest path

By definition, the average weighted shortest path [18] of the graph *G*_*k*_ is given by

$$\begin{array}{c} \lambda_{k}=\frac{\Lambda_{k}}{N_{k}\left(N_{k}-1\right)}, \end{array}$$ (1)

where

$$\begin{array}{c} \Lambda_{k}=\sum_{i,j\in G_{k}}p_{ij}^{\left(k\right)}, \end{array}$$ (2)

being $p_{ij}^{\left(k\right)}$ the weight shortest path linking nodes *i* and *j* in *G*_*k*_. Taking advantage of the recursive construction, we can decompose the sum Λ_*k*_ into two terms:

$$\begin{array}{c} \Lambda_{k}=\left(1+r\right)\Lambda_{k-1}+\Omega_{k-1}, \end{array}$$ (3)

where the first contribution takes into account all paths starting from and arriving at nodes belong to the same $G_{k-1}^{\left(\alpha \right)}(\alpha =0,1,2,\cdots ,M)$. Using the scaling mechanism for the edges, the first contribution can be easily identified with (1+*r*)Λ_*k*−1_. The second term takes into account all paths starting from and arriving at nodes belong to the different $G_{k-1}^{\left(\alpha \right)}(\alpha =0,1,2,\cdots ,M)$. Note that the paths that contribute to Ω_*k*−1_ must all go through at least hub node *A* in *G*_*k*_. The analytical expression for Ω_*k*−1_, called the length of crossing paths, is found below.

Denote $\Omega_{k-1}^{\alpha ,\beta }$ as the sum of length for all shortest paths with starting from nodes belong to $G_{k-1}^{\left(\alpha \right)}$ and arriving at nodes belong to $G_{k-1}^{\left(\beta \right)}$, respectively. Then the sum Ω_*k*−1_ is

$$\begin{array}{ccc} \Omega_{k-1} & = & 2(\Omega_{k-1}^{1,0}+\Omega_{k-1}^{2,0}+\cdots +\Omega_{k-1}^{M,0}\\ & & +\;\Omega_{k-1}^{2,1}+\Omega_{k-1}^{3,1}+\cdots +\Omega_{k-1}^{M,1}\\ & & +\;\cdots +\Omega_{k-1}^{M,M-1}). \end{array}$$

In order to find $\Omega_{k-1}^{\alpha ,\beta }$, we define

$$\begin{array}{ccc} \Delta_{k-1}^{\left(0\right)} & = & \sum_{i\in G_{k-1}^{\left(0\right)},i\neq A}p_{iA}^{\left(k\right)},\\ \Delta_{k-1}^{\left(\alpha \right)} & = & \sum_{i\in G_{k-1}^{\left(\alpha \right)}}p_{iA}^{\left(k\right)},\;\;\alpha =1,2,\cdots ,M. \end{array}$$

Then

$$\begin{array}{ccc} \Omega_{k-1} & = & 2MN_{k-1}(\Delta_{k-1}^{\left(0\right)}\\ & & +\;\Delta_{k-1}^{\left(1\right)}+\cdots +\Delta_{k-1}^{\left(M\right)})\\ & = & 2M{\left(M+1\right)}^{k-1}\Delta_{k-1}, \end{array}$$ (4)

being $\Delta_{k-1}=\Delta_{k-1}^{\left(0\right)}+\Delta_{k-1}^{\left(1\right)}+\cdots +\Delta_{k-1}^{\left(M\right)}$.

Thus, the problem of determining Ω_*k*_ is reduced to find Δ_*k*_.

We can prove that

$$\begin{array}{ccc} \Delta_{k} & = & \frac{M^{2}-Mr+M+r}{M-r}{\left(M+1\right)}^{k}\\ & & -\;\frac{M+r}{M-r}{\left(r+1\right)}^{k}. \end{array}$$ (5)

It is obtained as follow.

According to the particular construction of the weighted hierarchical modular network, we can find that Δ_*k*_ obeys the following recursive relation. Then we have

(a) Δ_1_ = Δ_0_ + (*r* + *M*) + *M* ^2^, (see Fig. 1)
(b) Δ_2_ = Δ_1_ + *r*Δ_0_ + (*M* + 1)(*r* + *M*) + (*r* ^2^ + *M* ^2^) + *M* ^3^, (see Fig. 2)
(c) Δ_3_ = Δ_2_ + *r*Δ_1_ + *r* ^2^Δ_0_ + (*M* + 1)^2^(*r* + *M*) + (*M* + 1)(*r* ^2^ + *M* ^2^) + (*r* ^3^ + *M* ^3^) + *M* ^4^,
(d) Δ_4_ = Δ_3_ + *r*Δ_2_ + *r* ^2^Δ_1_ + *r* ^3^Δ_0_ + (*M* + 1)^3^(*r* + *M*) + (*M* + 1)^2^(*r* ^2^ + *M* ^2^) + (*M* + 1)(*r* ^3^ + *M* ^3^) + (*r* ^4^ + *M* ^4^) + *M* ^5^,
  ⋮
(e) Δ_*k*−2_ = Δ_*k*−3_ + *r*Δ_*k*−4_ + *r* ^2^Δ_*k*−5_ + ⋯ + *r* ^*k*−3^Δ_0_ + (*M* + 1)^*k*−3^(*r* + *M*) + (*M* + 1)^*k*−4^(*r* ^2^ + *M* ^2^) + ⋯ + (*r* ^*k*−2^ + *M* ^*k*−2^) + *M* ^*k*−1^,
(f) Δ_*k*−1_ = Δ_*k*−2_ + *r*Δ_*k*−3_ + *r* ^2^Δ_*k*−4_ + ⋯ + *r* ^*k*−2^Δ_0_ + (*M* + 1)^*k*−2^(*r* + *M*) + (*M* + 1)^*k*−3^(*r* ^2^ + *M* ^2^) + ⋯ + (*r* ^*k*−1^ + *M* ^*k*−1^) + *M* ^*k*^,
(g) Δ_*k*_ = Δ_*k*−1_ + *r*Δ_*k*−2_ + *r* ^2^Δ_*k*−3_ + ⋯ + *r* ^*k*−1^Δ_0_ + (*M* + 1)^*k*−1^(*r* + *M*) + (*M* + 1)^*k*−2^(*r* ^2^ + *M* ^2^) + ⋯ + (*r* ^*k*^ + *M* ^*k*^) + *M* ^*k* + 1^.

From the above equations, it is not difficult to have

${\left(r+1\right)}^{k-2}\left(\left(\text{b})-r\times (\text{a}\right)\right):$

$$\begin{array}{ccc} {\left(r+1\right)}^{k-2}\Delta_{2} & = & {r+1)}^{k-1}\Delta_{1}+{\left(r+1\right)}^{k-2}\left(r+M\right)\\ & & \times \;\left(M-r+1\right)+{\left(r+1\right)}^{k-2}r^{2}\\ & & +\;{\left(r+1\right)}^{k-2}M^{2}\left(M-r+1\right), \end{array}$$

${\left(r+1\right)}^{k-3}\left(\left(\text{c})-r\times (\text{b}\right)\right):$

$$\begin{array}{ccc} {\left(r+1\right)}^{k-3}\Delta_{3} & = & {\left(r+1\right)}^{k-2}\Delta_{2}\\ & & +\;{\left(r+1\right)}^{k-3}\left(M+1\right)\left(r+M\right)\left(M-r+1\right)\\ & & +\;{\left(r+1\right)}^{k-3}\left(r^{2}+M^{2}\right)\left(M-r+1\right)\\ & & +\;{\left(r+1\right)}^{k-3}r^{3}+{\left(r+1\right)}^{k-3}M^{3}\left(M-r+1\right),\\ & & \;\vdots \end{array}$$

(r+1)((f)−r×(e)):

$$\begin{array}{ccc} \left(r+1\right)\Delta_{k-1} & = & {\left(r+1\right)}^{2}\Delta_{k-2}\\ & & +\;\left(r+1\right){\left(M+1\right)}^{k-3}\left(r+M\right)\left(M-r+1\right)\\ & & +\;\left(r+1\right){\left(M+1\right)}^{k-4}\left(r^{2}+M^{2}\right)\left(M-r+1\right)\\ & & +\;\cdots +\left(r+1\right)\left(r^{k-2}+M^{k-2}\right)\left(M-r+1\right)\\ & & +\;\left(r+1\right)R^{k-1}+\left(r+1\right)M^{k-1}\left(M-r+1\right), \end{array}$$

(g)−*r* × (f):

$$\begin{array}{ccc} \Delta_{k} & = & \left(r+1\right)\Delta_{k-1}+{\left(M+1\right)}^{k-2}\left(r+M\right)\left(M-r+1\right)\\ & & +\;{\left(M+1\right)}^{k-3}\left(r^{2}+M^{2}\right)\left(M-r+1\right)+\cdots \\ & & +\;\left(M-r+1\right)\left(M+1\right)\left(r^{k-2}+M^{k-2}\right)\\ & & +\;\left(M-r+1\right)\left(r^{k-1}+M^{k-1}\right)\\ & & +\;r^{k}+M^{k}\left(M-r+1\right). \end{array}$$

Adding up these equations, we have

$$\begin{array}{ccc} \Delta_{k} & = & {\left(r+1\right)}^{k-1}\Delta_{1}\\ & & +\;\{\left(r+M\right)\left(M-r+1\right)[{\left(M+1\right)}^{k-2}\\ & & +\;{\left(M+1\right)}^{k-3}\left(r+1\right)+\\ & & \;\cdots +\left(M+1\right){\left(r+1\right)}^{k-3}+{\left(r+1\right)}^{k-2}]\\ & & +\;\left(r^{2}+M^{2}\right)\left(M-r+1\right)[{\left(M+1\right)}^{k-3}\\ & & +\;{\left(M+1\right)}^{k-4}\left(r+1\right)+\cdots +\left(M+1\right){\left(r+1\right)}^{k-4}\\ & & +\;{\left(r+1\right)}^{k-3}]+\cdots +\\ & & \;\left(r^{k-2}+M^{k-2}\right)\left(M-r+1\right)\left[\left(M+1\right)+\left(r+1\right)\right]\\ & & +\;\left(r^{k-1}+M^{k-1}\right)\left(M-r+1\right)\}\\ & & +\;[r^{k}+r^{k-1}\left(r+1\right)+\cdots \\ & & +\;r^{3}{\left(r+1\right)}^{k-3}+r^{2}{\left(r+1\right)}^{k-2}]\\ & & +\;\left(M-r+1\right)[M^{k}+M^{k-1}\left(r+1\right)+\cdots \\ & & +\;M^{3}{\left(r+1\right)}^{k-3}+M^{2}{\left(r+1\right)}^{k-2}]. \end{array}$$

Using Δ_1_ = *M* + (*r* + *M*) + *M* ^2^ = *M* ^2^ + 2*M* + *r*. So we have

$$\begin{array}{ccc} \Delta_{k} & = & \left(M^{2}+2M+r\right){\left(r+1\right)}^{k-1}\\ & & +\;\frac{M-r+1}{r-M}\{\left(r+M\right)\left[{\left(r+1\right)}^{k-1}-{\left(M+1\right)}^{k-1}\right]\\ & & +\;\left(r^{2}+M^{2}\right)\left[{\left(r+1\right)}^{k-2}-{\left(M+1\right)}^{k-2}\right]\\ & & +\;\cdots +\left(r^{k-2}+M^{k-2}\right)\left[{\left(r+1\right)}^{2}-{\left(M+1\right)}^{2}\right]\\ & & +\;\left(r^{k-1}+M^{k-1}\right)\left[\left(r+1\right)-\left(M+1\right)\right]\}\\ & & +\;r^{2}\left[{\left(r+1\right)}^{k-1}-r^{k-1}\right]\\ & & +\;\frac{\left(M-r+1\right)M^{2}}{r-M+1}\left[{\left(r+1\right)}^{k-1}-M^{k}\right] \end{array}$$

$$\begin{array}{ccc} & = & \left(M^{2}+2M+r\right){\left(r+1\right)}^{k-1}\\ & & +\;\frac{M-r+1}{r-M}{\{[r\left(r+1\right)}^{k-1}\\ & & +\;r^{2}{\left(r+1\right)}^{k-2}+\cdots +r^{k-1}\left(r+1\right)]\\ & & +\;[M{\left(r+1\right)}^{k-1}+M^{2}{\left(r+1\right)}^{k-2}\\ & & +\;\cdots +M^{k-1}\left(r+1\right)]\\ & & -\;[r{\left(M+1\right)}^{k-1}+r^{2}{\left(M+1\right)}^{k-2}\\ & & +\;\cdots +r^{k-1}\left(M+1\right)]\\ & & -\;[M{\left(M+1\right)}^{k-1}+M^{2}{\left(M+1\right)}^{k-2}\\ & & +\;\cdots +M^{k-1}\left(M+1\right)]\}\\ & & +\;r^{2}\left[{\left(r+1\right)}^{k-1}-r^{k-1}\right]\\ & & +\;\frac{\left(M-r+1\right)M^{2}}{r-M+1}\left[{\left(r+1\right)}^{k-1}-M^{k}\right]\\ & = & \frac{M^{2}-Mr+M+r}{M-r}{\left(M+1\right)}^{k}-\frac{M+r}{M-r}{\left(r+1\right)}^{k}. \end{array}$$

Inserting the obtained result for Δ_*k*_ given in Equation (5) into Equation (4), we obtain

$$\begin{array}{ccc} \Omega_{k} & = & \frac{2M(M^{2}-Mr+M+r)}{M-r}{\left(M+1\right)}^{2k}\\ & & -\;\frac{2M(M+r)}{M-r}{\left(r+1\right)}^{k}{\left(M+1\right)}^{k}. \end{array}$$ (6)

Considering the initial condition, from Equation (3) and Equation (6), we can obtain

$$\begin{array}{ccc} \Lambda_{k} & = & \left(M+1\right)M{\left(1+r\right)}^{k-1}+\Omega_{k-1}\\ & \; & \left(1+r\right)\Omega_{k-2}+\cdots +{\left(1+r\right)}^{k-2}\Omega_{1} \end{array}$$

$$\begin{array}{ccc} & = & \left(M+1\right)M{\left(1+r\right)}^{k-1}\\ & & +\;\frac{2M(M^{2}-Mr+M+r)}{M-r}\\ & & \times \;[{\left(M+1\right)}^{2(k-1)}+\left(1+r\right){\left(M+1\right)}^{2(k-2)}\\ & & +\;\cdots +{\left(1+r\right)}^{k-2}{\left(M+1\right)}^{2}]\\ & & -\;\frac{2M(M+r)}{M-r}{[\left(r+1\right)}^{k-1}{\left(M+1\right)}^{k-1}\\ & & +\;\left(1+r\right){\left(r+1\right)}^{k-2}{\left(M+1\right)}^{k-2}\\ & & +\;\cdots +{\left(1+r\right)}^{k-2}\left(r+1\right)\left(M+1\right)] \end{array}$$

$$\begin{array}{ccc} & = & \left(M+1\right)M{\left(1+r\right)}^{k-1}\\ & & +\;\frac{2M(M^{2}-Mr+M+r)}{M-r}\\ & \; & \cdot \frac{{\left(M+1\right)}^{2}\left[{\left(M+1\right)}^{2k-2}-{\left(1+r\right)}^{k-1}\right]}{M^{2}+2M-r}\\ & & -\;\frac{2\left(M+r\right)\left(M+1\right){\left(r+1\right)}^{k-1}}{M-r}\\ & \; & \cdot [{\left(M+1\right)}^{k-1}-1] \end{array}$$

$$\begin{array}{ccc} & = & \frac{2M(M^{2}-Mr+M+r)}{\left(M-r\right)\left(M^{2}+2M-r\right)}{\left(M+1\right)}^{2k}\\ & & -\;\frac{2(M+r)}{\left(M-r)(r+1\right)}{\left[\left(M+1\right)\left(r+1\right)\right]}^{k}\\ & & +\;\frac{-M^{4}-M^{3}+2M^{2}-M^{2}r+Mr+2M+2r}{M^{2}+2M-r}\\ & \; & \times {\left(r+1\right)}^{k-1}. \end{array}$$

Therefore

$$\begin{array}{ccc} \lambda_{k} & = & \frac{2M(M^{2}-Mr+M+r)}{\left(M-r\right)\left(M^{2}+2M-r\right)}\times \frac{{\left(M+1\right)}^{k}}{{\left(M+1\right)}^{k}-1}\\ & & -\;\frac{2(M+r)}{\left(M-r)(r+1\right)}\times \frac{{\left(r+1\right)}^{k}}{{\left(M+1\right)}^{k}-1}\\ & & +\;\frac{-M^{4}-M^{3}+2M^{2}-M^{2}r+Mr+2M+2r}{\left(r+1\right)\left(M^{2}+2M-r\right)\left[{\left(M+1\right)}^{k}-1\right]}\\ & \; & \times {\left(\frac{r+1}{M+1}\right)}^{k}, \end{array}$$ (7)

which provides the following asymptotic behavior in the limit of large *k*, when *k* → ∞

$$\begin{array}{c} \lambda_{k}\to \frac{2M(M^{2}-Mr+M+r)}{\left(M-r\right)\left(M^{2}+2M-r\right)}. \end{array}$$ (8)

Thus the network grows unbounded but with the logarithm of the network size, while the weighted shortest distances stay bounded. In other words, in the infinite network order (which implies infinite number of nodes), the average weighted shortest path tends to a constant value which depends only on the size of the original graph and the weight.

### Average shortest path

In the preceding text we have studied the average weighted shortest path on the non-homogeneous weighted hierarchical modular network. Now we can also compute the average shortest path, *l*_*k*_, formally obtained by setting *r*_1_ = *r*_2_ = ⋯ = *r*_*M*_ = 1 in the previous formulas Equation (1) and Equation (2). Hence

$$\begin{array}{ccc} \Delta_{k} & = & \left(M^{2}+3M\right){\left(M+1\right)}^{k-1}\\ & & +\;[2M\left(k-1\right){\left(M+1\right)}^{k-2}\\ & & +\;2M^{2}\left(k-2\right){\left(M+1\right)}^{k-3}+\cdot \cdot \cdot +2M^{k-2}\\ & \; & \cdot 2\left(M+1\right)+2M^{k-1}]+2[M^{k}+M^{k-1}\left(M+1\right)\\ & & +\;\cdot \cdot \cdot +M^{2}{\left(M+1\right)}^{k-2}]\\ & = & \left(M^{2}+3M\right){\left(M+1\right)}^{k-1}\\ & & +\;2M[\left(k-1\right){\left(M+1\right)}^{k-2}\\ & & +\;\left(k-2\right)M{\left(M+1\right)}^{k-3}+\cdot \cdot \cdot \\ & & +\;2M^{k-3}\left(M+1\right)+M^{k-2}]\\ & & +\;2M^{2}\left[{\left(M+1\right)}^{k-1}-M^{k-1}\right] \end{array}$$

We make

$$\begin{array}{ccc} Z & = & \left(k-1\right){\left(M+1\right)}^{k-2}+\left(k-2\right)M{\left(M+1\right)}^{k-3}\\ & \;\;\;\; & +\cdot \cdot \cdot +2M^{k-3}\left(M+1\right)+M^{k-2}, \end{array}$$

thus we obtain the expression of *Z* as

$$\begin{array}{c} Z=\left(k-M-1\right){\left(M+1\right)}^{k-1}+M^{k}, \end{array}$$

Then, from the above equations we get the following relation

$$\begin{array}{c} \Delta_{k}=M\left(2k+M+1\right){\left(M+1\right)}^{k-1}. \end{array}$$

So, we can get

$$\begin{array}{c} \Omega_{k}=2M{\left(M+1\right)}^{k}\Delta_{k}=2M^{2}\left(2k+M+1\right){\left(M+1\right)}^{2k-1}, \end{array}$$

Taking the initial condition Λ_1_ = (*M*+1)*M* into account, we obtain

$$\begin{array}{ccc} \Lambda_{k} & = & \left(M+1\right)M{\left(1+M\right)}^{k-1}+\Omega_{k-1}\\ & & +\;\left(1+M\right)\Omega_{k-2}+\cdots \\ & & +\;{\left(1+M\right)}^{k-2}\Omega_{1}\\ & = & M{\left(M+1\right)}^{k}+2M^{2}(\left(2\left(k-1\right)+M+1\right)\\ & \; & \times {\left(M+1\right)}^{2k-3}\\ & & +\;\left(1+M\right)\left(2\left(k-2\right)+M+1\right){\left(M+1\right)}^{2k-5}\\ & & +\;\cdots +{\left(M+1\right)}^{k-2}\left(2+M+1\right)\left(M+1\right))\\ & = & M{\left(M+1\right)}^{k}+2M^{2}{[2\left(k-1\right)\left(M+1\right)}^{2k-3}\\ & & +\;2\left(k-2\right){\left(M+1\right)}^{2k-4}\\ & & +\;\cdot \cdot \cdot +2{\left(M+1\right)}^{k-1}]\\ & & +\;2M^{2}{[\left(M+1\right)}^{2k-2}+{\left(M+1\right)}^{2k-3}\\ & & +\;\cdot \cdot \cdot +{\left(M+1\right)}^{k}]\\ & = & M{\left(M+1\right)}^{k}+4M^{2}H+2M{\left(M+1\right)}^{2k-1}\\ & & -\;2M{\left(M+1\right)}^{k}\\ & = & 2M{\left(M+1\right)}^{2k-1}-M{\left(M+1\right)}^{k}+4M^{2}H \end{array}$$

In the above expression,

$$\begin{array}{ccc} H & = & \left(k-1\right){\left(M+1\right)}^{2k-3}+\left(k-2\right){\left(M+1\right)}^{2k-4}\\ & & +\;\cdot \cdot \cdot +{\left(M+1\right)}^{k-1}\\ & = & \frac{kM-M-1}{M^{2}}{\left(M+1\right)}^{2k-2}+\frac{1}{M^{2}}{\left(M+1\right)}^{k-1} \end{array}$$

so, we get the result

$$\begin{array}{ccc} \Lambda_{k} & = & 2\left(2Mk+M^{2}-M-2\right){\left(M+1\right)}^{2k-2}\\ & & -\;\left(M^{2}+M-4\right){\left(M+1\right)}^{k-1}. \end{array}$$

Therefore,

$$\begin{array}{ccc} l_{k} & = & \frac{2(2Mk+M^{2}-M-2)}{{\left(M+1\right)}^{2}}\times \frac{{\left(M+1\right)}^{k}}{{\left(M+1\right)}^{k}-1}\\ & & -\;\frac{M^{2}+M-4}{M+1}\times \frac{1}{{\left(M+1\right)}^{k}-1}, \end{array}$$ (9)

which provides the following asymptotic behavior, when *k* → ∞

$$\begin{array}{ccc} l_{k} & ∼ & \frac{2(2Mk+M^{2}-M-2)}{{\left(M+1\right)}^{2}} \end{array}$$ (10)

$$\begin{array}{ccc} & ∼ & \frac{4M}{{\left(M+1\right)}^{2}\ln\left(M+1\right)}\ln N_{k},\;\;k\to \infty . \end{array}$$ (11)

From the above quantity, the average shortest path grows as the logarithm of the total number of nodes. T. Carletti and S. Righi [4] have defined a class of weighted fractal networks. The average shortest path, *l*_*k*_ of weighted fractal networks grows as the logarithm of the total number of nodes.

**Fig 3:**
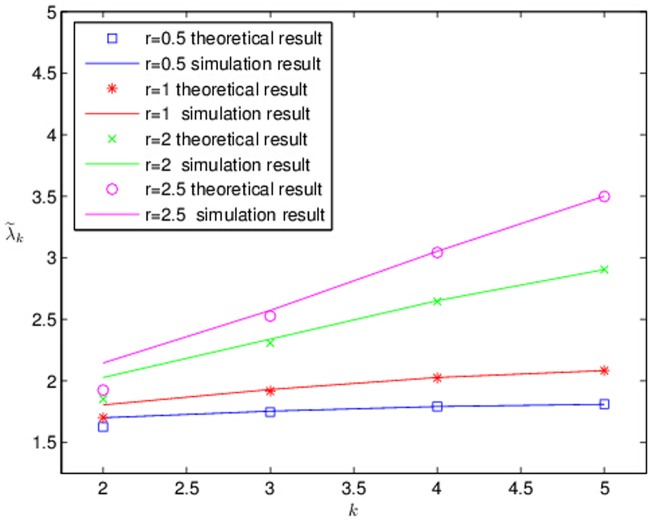
The comparisons of theoretical predictions and simulation results the renormalized average weighted shortest path $\tilde{\lambda_{k}}$ of *λ*_*k*_. versus the iteration, where *M* = 3 and ${\tilde{\lambda }}_{k}=\frac{\lambda_{k}-\min\{\lambda_{k}\}}{\max\{\lambda_{k}\}-\min\{\lambda_{k}\}}$. Under Floyd algorithm, data points are obtained by simulating the network at generation *k* = 2,3,4,5, respectively.

We have made the comparisons of theoretical predictions and simulation results of the average weighted shortest path. Under Floyd algorithm, data points are obtained by simulating the network at generation *k* = 2,3,4,5 respectively (see Fig. 3).

## Conclusions

In this paper, we have introduced a non-homogeneous weighted hierarchical modular network and related it to the average weighted shortest path. In addition, we have already compared the relation between the average weighted shortest path and the average shortest path. That is to say, in the infinite network order implying infinite number of nodes, the average weighted shortest path tends to a constant value which depends only on the size of the original graph and the weight. However, the average shortest path grows as the logarithm of the total number of nodes. We built weighted hierarchical modular networks in an iterative way. In fact the average shortest path increases logarithmically with the system size. Moreover the average weighted shortest path in turn depends on the number of copies and the weighted scaling factors. In other words the topology of such networks could have been shaped by evolution in such a way that any two nodes can be connected in a finite optimal time, whatever their physical distance is.
